# Impact of effective refractory period personalization on arrhythmia vulnerability in patient-specific atrial computer models

**DOI:** 10.1093/europace/euae215

**Published:** 2024-08-23

**Authors:** Patricia Martínez Díaz, Albert Dasí, Christian Goetz, Laura A Unger, Annika Haas, Armin Luik, Blanca Rodríguez, Olaf Dössel, Axel Loewe

**Affiliations:** Department of Electrical Engineering and Information Technology, Institute of Biomedical Engineering, Karlsruhe Institute of Technology (KIT), Fritz-Haber, Weg 1 30.33, 76131, Karlsruhe, Germany; Department of Computer Science, University of Oxford, 7 Parks Rd, OX13QG, Oxford, England, UK; Department of Electrical Engineering and Information Technology, Institute of Biomedical Engineering, Karlsruhe Institute of Technology (KIT), Fritz-Haber, Weg 1 30.33, 76131, Karlsruhe, Germany; Department of Cardiology, University Hospital Heidelberg, Im Neuenheimer Feld 410, 69120, Heidelberg, Germany; Department of Electrical Engineering and Information Technology, Institute of Biomedical Engineering, Karlsruhe Institute of Technology (KIT), Fritz-Haber, Weg 1 30.33, 76131, Karlsruhe, Germany; Medizinische Klinik IV, Städtisches Klinikum Karlsruhe, Teaching Hospital of the University of Freiburg, Moltkestraße 90, 76133, Karlsruhe, Germany; Medizinische Klinik IV, Städtisches Klinikum Karlsruhe, Teaching Hospital of the University of Freiburg, Moltkestraße 90, 76133, Karlsruhe, Germany; Medizinische Klinik IV, Städtisches Klinikum Karlsruhe, Teaching Hospital of the University of Freiburg, Moltkestraße 90, 76133, Karlsruhe, Germany; Department of Computer Science, University of Oxford, 7 Parks Rd, OX13QG, Oxford, England, UK; Department of Electrical Engineering and Information Technology, Institute of Biomedical Engineering, Karlsruhe Institute of Technology (KIT), Fritz-Haber, Weg 1 30.33, 76131, Karlsruhe, Germany; Department of Electrical Engineering and Information Technology, Institute of Biomedical Engineering, Karlsruhe Institute of Technology (KIT), Fritz-Haber, Weg 1 30.33, 76131, Karlsruhe, Germany

**Keywords:** Effective refractory period, Atrial fibrillation, Computer model, Arrhythmia vulnerability, Digital twin

## Abstract

**Aims:**

The effective refractory period (ERP) is one of the main electrophysiological properties governing arrhythmia, yet ERP personalization is rarely performed when creating patient-specific computer models of the atria to inform clinical decision-making. This study evaluates the impact of integrating clinical ERP measurements into personalized *in silico* models on arrhythmia vulnerability.

**Methods and results:**

Clinical ERP measurements were obtained in seven patients from multiple locations in the atria. Atrial geometries from the electroanatomical mapping system were used to generate personalized anatomical atrial models. The Courtemanche M. *et al.* cellular model was adjusted to reproduce patient-specific ERP. Four modeling approaches were compared: homogeneous (A), heterogeneous (B), regional (C), and continuous (D) ERP distributions. Non-personalized approaches (A and B) were based on literature data, while personalized approaches (C and D) were based on patient measurements. Modeling effects were assessed on arrhythmia vulnerability and tachycardia cycle length, with sensitivity analysis on ERP measurement uncertainty. Mean vulnerability was 3.4 ± 4.0%, 7.7 ± 3.4%, 9.0 ± 5.1%, and 7.0 ± 3.6% for scenarios A–D, respectively. Mean tachycardia cycle length was 167.1 ± 12.6 ms, 158.4 ± 27.5 ms, 265.2 ± 39.9 ms, and 285.9 ± 77.3 ms for scenarios A–D, respectively. Incorporating perturbations to the measured ERP in the range of 2, 5, 10, 20, and 50 ms changed the vulnerability of the model to 5.8 ± 2.7%, 6.1 ± 3.5%, 6.9 ± 3.7%, 5.2 ± 3.5%, and 9.7 ± 10.0%, respectively.

**Conclusion:**

Increased ERP dispersion had a greater effect on re-entry dynamics than on vulnerability. Inducibility was higher in personalized scenarios compared with scenarios with uniformly reduced ERP; however, this effect was reversed when incorporating fibrosis informed by low-voltage areas. Effective refractory period measurement uncertainty up to 20 ms slightly influenced vulnerability. Electrophysiological personalization of atrial *in silico* models appears essential and requires confirmation in larger cohorts.

## Graphical Abstract

**Graphical Abstract:**
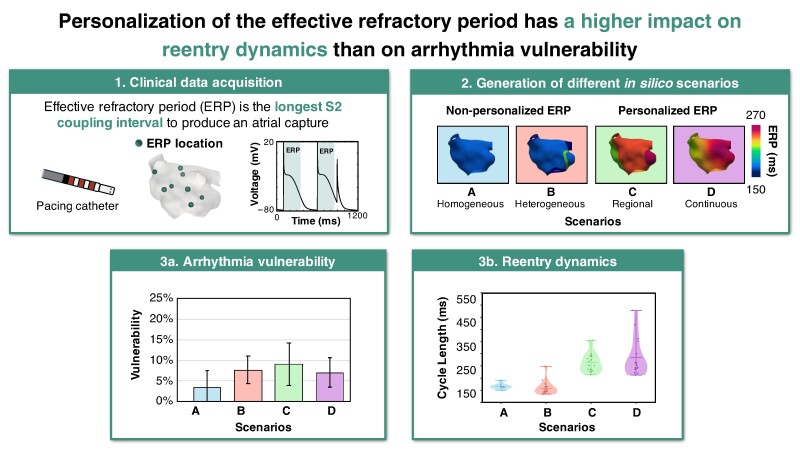

## Introduction

Refractoriness is an electrophysiological property that characterizes the response of cardiac tissue to premature stimulation. Shortened cardiac refractoriness promotes sustained re-entrant activity^1^ and can be assessed during electrophysiological studies following the extra-stimulus S1S2 pacing technique, where a train of S1 stimuli is given at a certain cycle length followed by a premature S2 stimulus.^2^ The effective refractory period (ERP) can then be defined as the longest S1S2 interval that fails to generate a capture in the tissue. Refractory period can only be determined at one region at a time (between stimulus and measurement locations); thus, multiple measurements are necessary for estimations of spatial distribution.^1^

Although ERP is often linked to the action potential duration (APD), this relationship is inconsistent, particularly in the presence of structural abnormalities.^3,4^ Effective refractory period can be influenced by the stimulus type and the local structural environment, such as electrotonic loading. This makes ERP especially relevant in cases of fibrosis, as fibrosis can affect ERP without significantly altering APD.

Clinical and pre-clinical investigations have demonstrated heterogeneous refractoriness properties across different atrial regions, which also vary from patient to patient.^5−7^ During atrial fibrillation (AF), high stimulation frequencies induce electrical remodeling, resulting in shortened APD and ERP.^8^ However, contrary to the belief that prolonged exposure to AF always shortens ERP, patients with persistent AF may exhibit longer ERP due to the presence of atrial dilatation.^4^ So, the overall contribution of refractoriness to increased re-entrant inducibility remains unclear.

A common theory explaining the existence of AF postulates that both a trigger and a vulnerable substrate are necessary for the initiation and maintenance of AF.^9^ Ectopic activity from the sleeves of the pulmonary veins (PVs) is the most frequent form of AF triggers.^10^ Non-PV triggers have been identified in the crista terminalis (CT), the interatrial septum, the left atrium (LA) posterior wall, the left atrial appendage (LAA), the ligament of Marshall, the superior vena cava (SVC), and the coronary sinus, yet their precise role in initiating AF remains uncertain.^11^ The vulnerable substrate refers to changes in electrical and structural remodeling (e.g. shortening of the APD, presence of fibrosis, atrial dilatation, adipose tissue infiltration, and inflammation).^12^ The presence of electrical heterogeneity, such as regional variations in conduction velocity (CV), APD, and ERP, can favour unidirectional block in response to ectopy/stimulation, which can then initiate re-entry.^13^ However, it is still challenging to characterize the vulnerable substrate in a clinical or experimental setting. Understanding the interplay between electrophysiological and structural factors and how their regional distribution (heterogeneity) influences arrhythmia maintenance remains a complex task in cardiac electrophysiology research.

In this sense, patient-specific atrial computational models provide a robust framework for studying, under controlled conditions, the integrated effect of substrate features unique to each patient and their impact on arrhythmia vulnerability.^14−16^ The creation of patient-specific computer models of the atria typically involves anatomical personalization using image data obtained from magnetic resonance imaging (MRI), computed tomography scans, or electroanatomic mapping systems (EAMS). Electrophysiological personalization is rarely performed since patient electrophysiological data are usually not available beforehand.^17^ Some studies have conducted personalization of atrial electrophysiology, by fitting model parameters to patient clinical data.^14,17−20^ Their findings suggest that personalized electrophysiological parameter values vary among patients and differ from standardized literature parameter values. Nevertheless, the effect of incorporating patient-specific clinical ERP measurements on arrhythmia vulnerability has not yet been assessed. In this work, we investigate the role of incorporating personalized ERP values from various clinical measurements on the *in silico* assessment of arrhythmia vulnerability.

## Methods

### Electrophysiological study

Six patients with a history of AF and prior pulmonary vein isolation (PVI) and one patient with atrial flutter (AFl) underwent an electrophysiological study. Patients gave written informed consent, and the ethics committee of Städtisches Klinikum Karlsruhe gave ethical approval for this work. Electroanatomic maps during sinus rhythm were generated using the Rhythmia 3D mapping system (Boston Scientific, USA). For patients with prior PVI, LA mapping was conducted, whereas for the patient with AFl, the right atrium (RA) was mapped. Effective refractory period measurements were obtained from multiple locations in the atria (5.7 ± 1.4 measurements) following an S1S2 protocol with seven S1 stimuli at a basic cycle length of 500 ms and an S2 stimulus with intervals between 300 and 200 ms, decreasing by 10 ms until loss of capture. Pacing stimuli for clinical ERP identification had an amplitude of 5 V with a duration of 1 ms in a bipolar configuration, using either the Intellamap Orion™ 8.5 F catheter or the Intellanav Stablepoint™ 7 F catheter (Boston Scientific, USA). Stimulus capture was verified for each location, and the amplitude was incrementally increased at locations where no capture was achieved initially.

**Figure 1:**
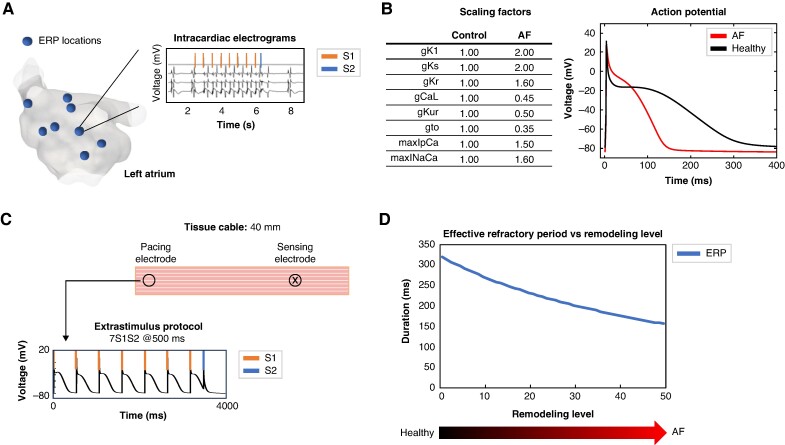
Modeling of patient-specific effective refractory period. *A*) ERP was measured in multiple locations in the atria with an S1S2 protocol. *B*) Scaling of the maximum ion channel conductances of the Courtemanche M. *et al.* cellular computer model from control to AF. *C*) *In silico* pacing protocol in tissue cable. *D*) ERP with respect to remodeling level. AF, atrial fibrillation; ERP, effective refractory period.

Effective refractory period measurements were taken in different anatomical regions such as the anterior wall, posterior or lateral wall, appendage, and at least one PV in the case of LA geometries. A representative endocardial trace of the stimulation protocol is shown in *Figure 1A*. Effective refractory period was defined as the longest S1S2 interval without capture. To characterize the patient-specific fibrotic substrate, low-voltage areas (LVAs: *<*0.5 mV) were identified from bipolar voltage maps.

### Patient-specific anatomical modeling

The atrial anatomy derived from the EAMS was utilized to generate personalized simulation-ready bilayer meshes. The seven bilayer meshes were created using AugmentA^21^ including rule-based anatomical annotations and fiber orientations. For the LA models, the LAA, mitral valve, PV, and left Bachmann’s bundle (BB) were automatically annotated, and for the RA model, the tricuspid valve, right atrial appendage, SVC, inferior vena cava, pectinate muscles (PM), right BB, and CT. An open-source Python-based algorithm was used to subdivide the meshes into anatomical regions—anterior wall, septal wall, posterior wall, lateral wall, inferior wall, and appendage—for the RA and LA accordingly.^22^

### Atrial electrophysiology modeling

**Figure 2:**
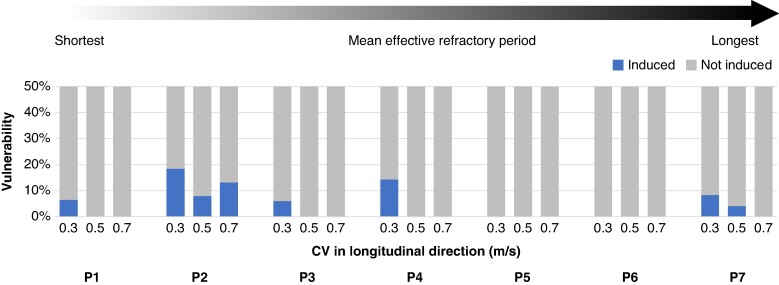
Comparison of arrhythmia vulnerability with non-personalized heterogeneous effective refractory period distribution (scenario B). A conduction velocity of 0.3 m/s revealed a higher number of inducing points in the control scenario. P1–P7 indicate individual patients. CV, conduction velocity; ERP, effective refractory period.

Electrical propagation in the atria was modeled using the monodomain equation and simulated with openCARP.^23^ Anisotropy in different parts of the atria was modeled as described in Krueger M. *et al*.^17^ Conduction velocity was doubled in the CT and tripled in the PM and in the BB.^24^ As both ERP and CV influence re-entry maintenance,^19^ our aim was to identify the CV at which vulnerability was highest. We tuned the longitudinal monodomain conductivity to achieve a mean CV of 0.3, 0.5, and 0.7 m/s in the bulk myocardium, for each patient-specific model. A CV of 0.3 m/s, as shown in *Figure 2*, exhibited the highest number of inducing points and was therefore selected for further vulnerability assessments. To reach a limit cycle, single-cell models were paced 100 times with a basic cycle length of 500 ms. Single-chamber models were paced four times from the earliest activation site identified from local activation maps, also with a basic cycle length of 500 ms.

### Patient-specific effective refractory period modeling

To reproduce patient-specific clinical ERP *in silico*, the maximum conductances of key ionic channels affecting action potential morphology in the established Courtemanche M. *et al.* cellular model^25^ were modified from control conditions to a setup that reflects changes in the action potential observed in patients with persistent AF (*Figure* *1B*).^24^ We modified the maximum conductances of the inward rectifier K^+^ current (*g*_K1_), the ultrarapid (*g*_Kur_), the rapid (*g*_Kr_), the slow delayed-rectifier (*g*_s_), and the transient outward (*g*_to_) K^+^ currents; the L-type Ca^2+^ current (*g*_CaL_), the sarcoplasmic Ca^2+^ pump current (*I*_pCa_), and the Ca^2+^/Na^+^ exchanger (max*I*_NaCa_). The ion channel conductances were linearly scaled to generate a set of 50 different cellular models with gradually increasing remodeling levels. We generated an *in silico* tissue cable for each cellular model with a length of 40 mm and a resolution of 0.4 mm and performed a virtual S1S2 pacing protocol to obtain the ERP (*Figure* *1C* and *D*). The scaling factors for each combination and the corresponding ERP can be found in Supplementary material online, *Table S1*. Pacing stimuli for *in silico* ERP identification had a current density of 30 *µ*A*/*cm^2^ with a duration of 3 ms and an S2 coupling interval ranging from 350 ms up to loss of capture in steps of 1 ms.

### Generation of effective refractory period scenarios

**Figure 3:**
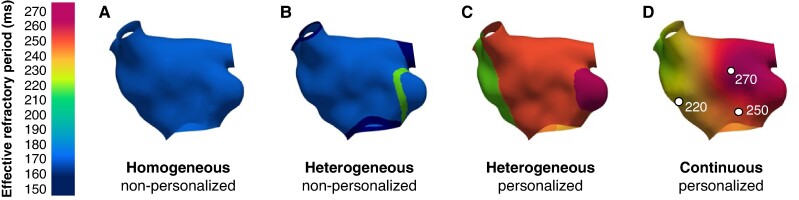
Scenarios for the evaluation of effective refractory period personalization. Homogeneous *A*) with non-personalized ERP based on literature data,^24^ heterogeneous *B*) with different non-personalized ERP based on anatomical structures,^17^ regional *C*) with personalized ERP divided into anatomical regions, and continuous *D*) with personalized ERP from interpolated measurements. Circles denote catheter tip locations where the pacing stimulus was delivered. ERP, effective refractory period.

To assess the role of ERP personalization, we generated four scenarios: homogeneous (A), heterogeneous (B), regional (C), and continuous (D) ERP distribution (*Figure 3*). The first two configurations were non-personalized based on literature data, and the latter two were personalized based on patient measurements. In scenario A, the same cellular model corresponding to AF-induced remodeling^24^ was applied to the whole atrium. In scenario B, anatomical structures had individual cellular model variants with specific ERP assigned based on literature data.^17^ For scenario C, all nodes in anatomical regions were assigned distinct cellular models with the ERP value matching the spatially closest available clinical measurement. In case of multiple measurements present in the same region, the average ERP value was considered for the whole region. In scenario D, the measured ERPs were assigned to the corresponding catheter tip positions and then continuously mapped to the whole surface by Laplacian interpolation.^26^ The measuring points were defined as boundary conditions, so that the ERP values never exceeded the measured ERP range. The ion channel conductances for each individual mesh node were then adjusted accordingly to match the interpolated ERP. For each modified ionic channel conductance in the Courtemanche M. *et al.* model, an adjustment file was generated, consisting of a list with the corresponding scaling factor for the ERP value at each node in the mesh. Adjustment files were generated in openCARP using the adjustment function as described in Boyle P.^27^ The four personalization scenarios are shown in *Figure 1*.

### Patient-specific substrate modeling

Substrate was incorporated into the meshes based on the identification of LVA. To distinguish between ablation lesions from PVI and native fibrosis, we defined ablation lesion regions as having voltage *<* 0.1 mV and native fibrosis regions as having a voltage between 0.1 and 0.5 mV.^13^ To model native fibrosis, we accounted for two different cellular mechanisms: replacement fibrosis and inflammation. For regions defined as native fibrosis, 30% of the elements were randomly selected and set to non-conductive with *σ* = 10^−7^ S/m to represent replacement fibrosis, while the remaining 70% were set to be electrically remodelled in response to cellular inflammation.^28,14^ Several ionic conductances were rescaled to represent the effects of electrical remodeling (*g*_CaL_ ×22.5%, *g*_Na_ ×60%, factor*g*_Kur_ ×50%, *g*_to_ ×35%, *g*_Ks_ ×200%, max*I*_pCa_ × 150%, max*I*_NaCa_ × 160%). To model ablation lesions, all elements were set to be non-conductive.^29^ To assess the influence of ablation lesions and native fibrosis on arrhythmia vulnerability, we created four additional scenarios, namely, A2, D2, A3, and D3. The first two scenarios, A2 and D2, included ablation lesions and native fibrosis. Scenario A2 had the same ERP as scenario A (homogeneous), and scenario D2 corresponding to same ERP personalization as scenario D (continuous). Lastly, to model a stage before PVI, we generated scenarios A3 and D3 including only native fibrosis regions, and ablation lesions were modeled as healthy.

### Vulnerability assessment

Arrhythmia vulnerability was assessed by virtual S1S2 pacing at different locations in the atria separated by an average distance of 2 cm.^30^ The vulnerability ratio was defined as the number of inducing points divided by the number of stimulation points. Stimulation point locations remained consistent among scenarios. Transmembrane voltage traces were recorded for 1 s for each re-entry at the inducing stimulus location. We determined the tachycardia cycle length (TCL) of the re-entries by calculating the average between peaks of d*V*/d*t*.

### Sensitivity analysis

To study the influence of uncertainty in ERP measurements, we conducted a sensitivity analysis by including perturbation in ERP measurements in the ranges of ±2, ±5, ±10, ±20, and ±50 ms, randomly drawn from a uniform distribution. We generated 10 perturbation sets for each perturbation range, resulting in a total of 50 new perturbed ERP sets; a separate random value was drawn for each measured ERP. Finally, we generated new interpolated maps using the perturbed ERP sets. Due to the high computational cost of the vulnerability assessment (15 ± 2.4 min per stimulation point, utilizing 4 nodes × 40 CPU cores with Intel Xeon Gold 62 302.1 GHz), the sensitivity analysis was limited to the assessment of the patient P3 model, which showed the highest vulnerability in the LA model cohort.

### Statistical analysis

The data are presented as mean ± SD. We used a two-sample *t*-test to determine statistical significance between the sample means. *P*  *<* 0.05 were considered significant.

## Results

**Figure 4:**
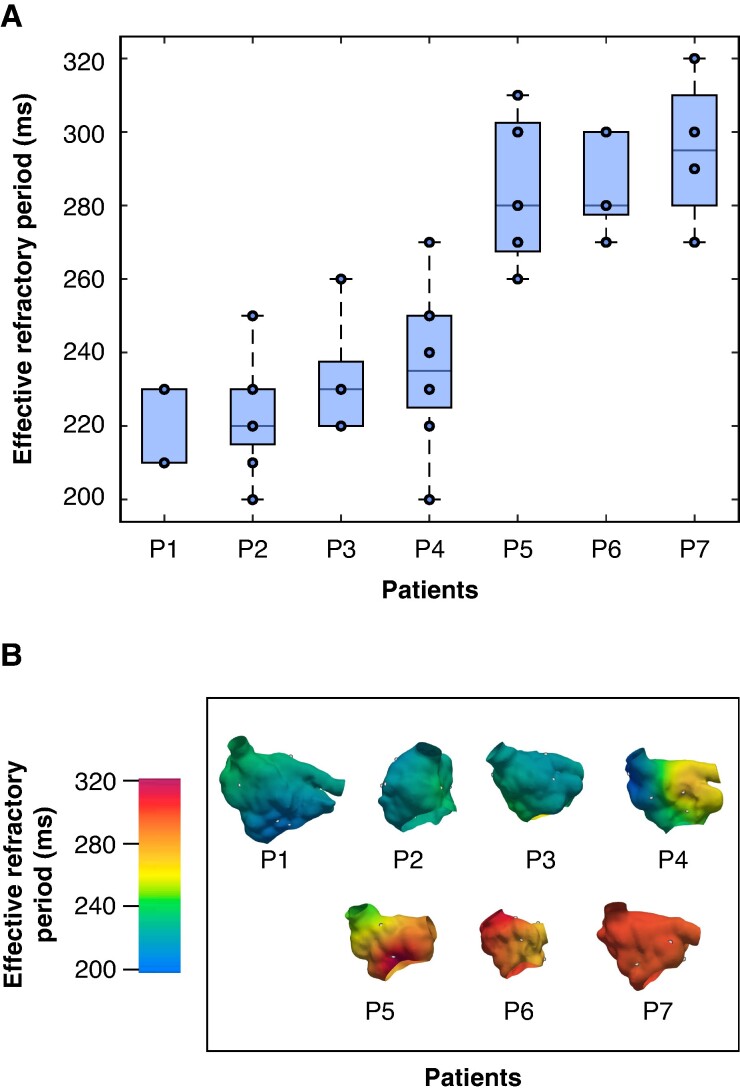
Distribution of clinically measured effective refractory period. *A*) Boxplots showing the dispersion of ERP measurements for each patient, where the points represent each individual measurement. *B*) ERP distribution maps generated from interpolated clinical measurements from an anterior view. P1–P7 indicate individual patients. ERP, effective refractory period.

**Figure 5:**
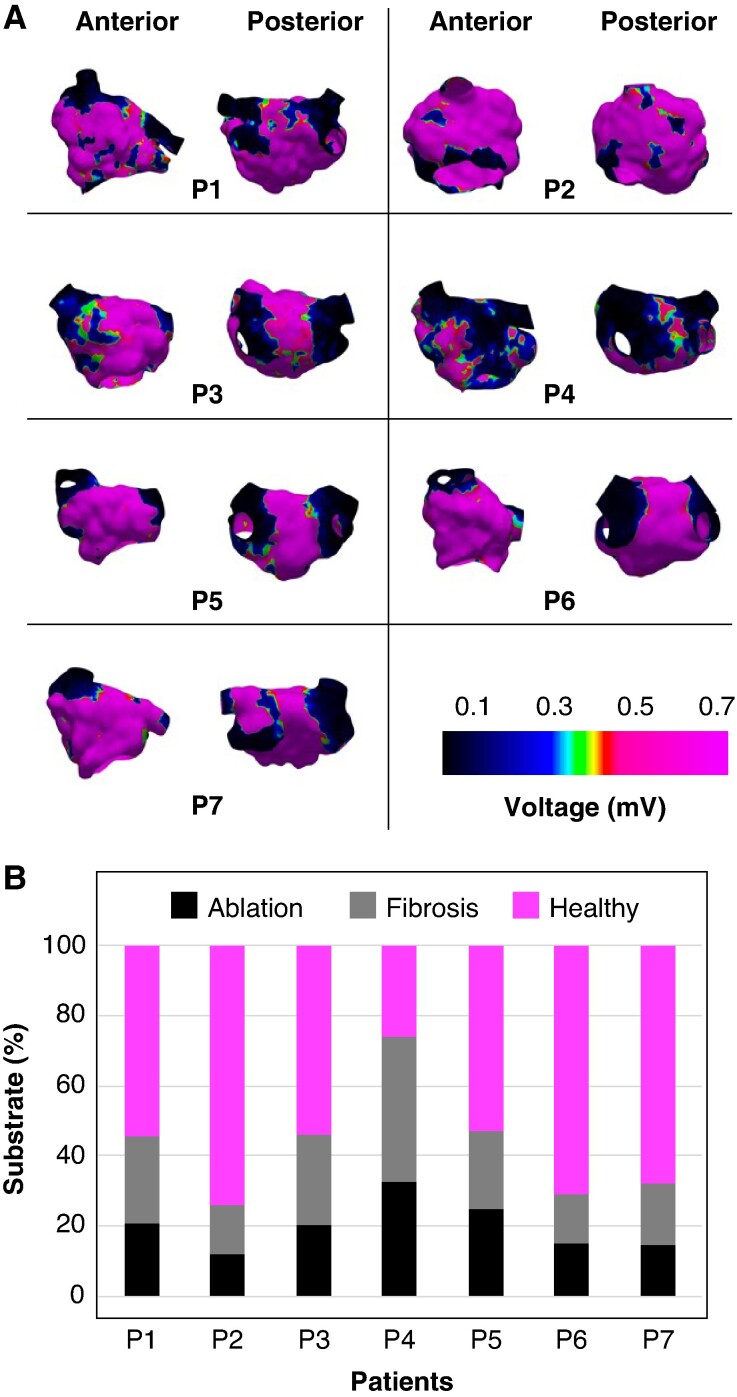
Distribution of substrate based on the identification of low-voltage areas. *A*) Bipolar voltage maps from anterior and posterior views. *B*) Percentage of substrate defined as ablation lesions (*<*0.1 mV), fibrotic regions (0.1–0.5 mV), and healthy tissue (*>*0.5 mV). P1–P7 indicate individual patients.

Patient characteristics are outlined in *Table 1*. The overall clinically measured ERP was 254.0 ± 32.7 ms. The dispersion of the ERP measurements is shown in the boxplot in *Figure 4A*. The ERP distribution maps for each patient are illustrated in *Figure 4B*. Bipolar voltage maps for each patient are shown in *Figure 5A*. Low-voltage areas accounted for 42.8 ± 16.4% of the atrial surface. The amount of fibrosis, ablation lesions, and healthy tissue for each patient is shown in *Figure 5B*.

**Table 1:** Clinical characteristics of patient cohort

Patient	Sex	Chamber	Volume (ml)	Area (*cm*^2^)	LVA (%)	ERP (ms)	ERP (#)	Dx
P1	F	LA	270.1	267.4	45.49	222.0 ± 11.0	5	PAF
P2	M	RA	221.4	200.7	25.78	222.5 ± 14.9	8	AFl
P3	F	LA	175.9	179.8	46.04	232.0 ± 16.4	7	PAF
P4	M	LA	175.6	187.4	74.02	236.3 ± 21.3	5	PeAF
P5	M	LA	86.9	120.0	47.36	284.0 ± 20.7	5	PeAF
P6	F	LA	95.2	121.2	28.85	286.0 ± 13.4	4	PAF
P7	M	LA	106.8	136.9	32.35	295.0 ± 20.8	6	PAF

Dx, diagnosis; ERP, effective refractory period; F, female; LA, left atrium; LVA, low-voltage area; M, male; PAF, paroxysmal atrial fibrillation; PeAF, persistent atrial fibrillation; RA, right atrium; #, number of measurements.

**Figure 6:**
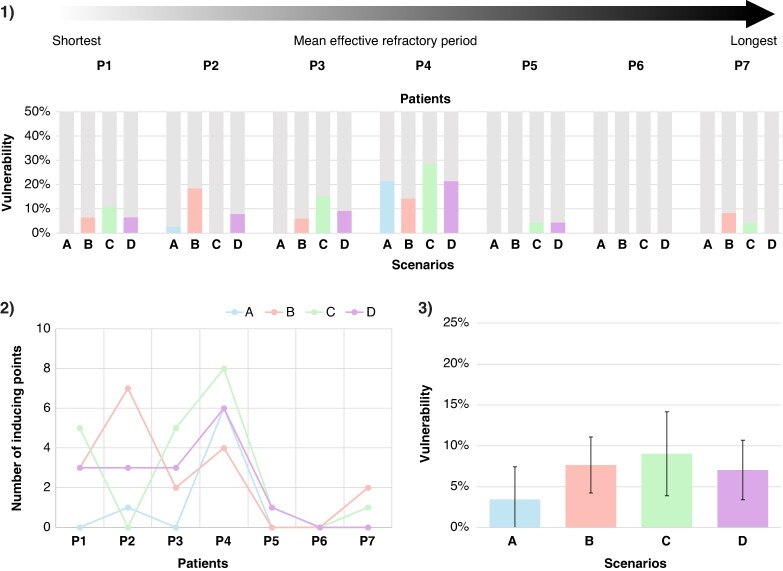
Comparison of arrhythmia vulnerability and number of inducing points among four scenarios. *1*) Vulnerability for each patient in four personalization scenarios without fibrotic substrate. *2*) Number of inducing points for each patient. *3*) Mean vulnerability; bars indicate standard deviation. P1–P7 indicate individual patients. Scenarios are defined as A, homogeneous; B, heterogeneous; C, regional; and D, continuous ERP distribution. ERP, effective refractory period.

**Figure 7:**
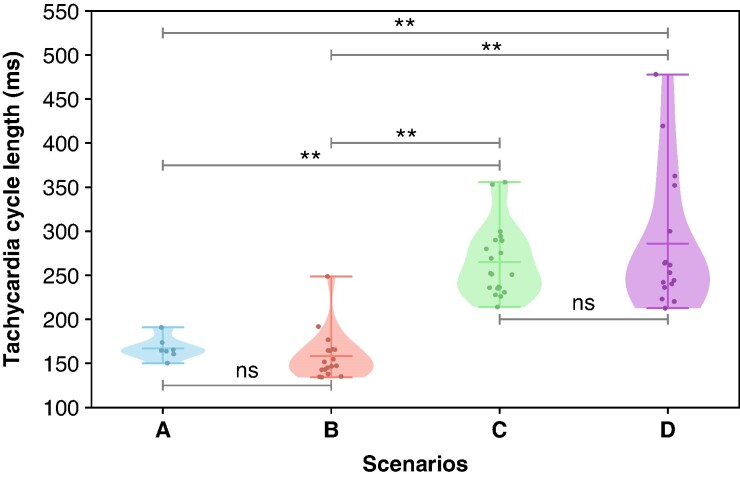
Tachycardia cycle length of induced re-entries. Each individual point represents one re-entry. Conduction velocity in the bulk myocardium was set to 0.3 m/s in the longitudinal direction. **P  *<* 0.05; ns, not statistically significant. Scenarios are defined as A, homogeneous; B, heterogeneous; C, regional; and D, continuous ERP distribution. ERP, effective refractory period.

The ERP of *in silico* tissue cables varied from 320 ms in the healthy state to 157 ms in the AF remodeling state. Non-personalized scenarios had a shorter ERP and reduced dispersion with an ERP of 158.9 ± 5.3 ms, while personalized scenarios had an ERP of 254.0 ± 32.7 ms. From a total of 214 stimulation points (30.6 ± 8.9 stimulation points per patient), 61 simulated re-entries were induced across the four scenarios without fibrotic substrate, with individual counts of 7, 18, 20, and 16 re-entries for scenarios A, B, C, and D, respectively. Vulnerability values are shown in *Figure 6*. The vulnerability for scenario A was 3.4 ± 4.0%, 7.7 ± 3.4% for scenario B, 9.0 ± 5.1% for scenario C, and 7.0 ± 3.6% for scenario D. The mean TCL was 167.07 ± 12.58 ms for scenario A, 158.42 ± 27.52 ms for scenario B, 265.17 ± 39.87 ms for scenario C, and 285.88 ± 77.31 ms for scenario D, as shown in *Figure 7*.

**Figure 8:**
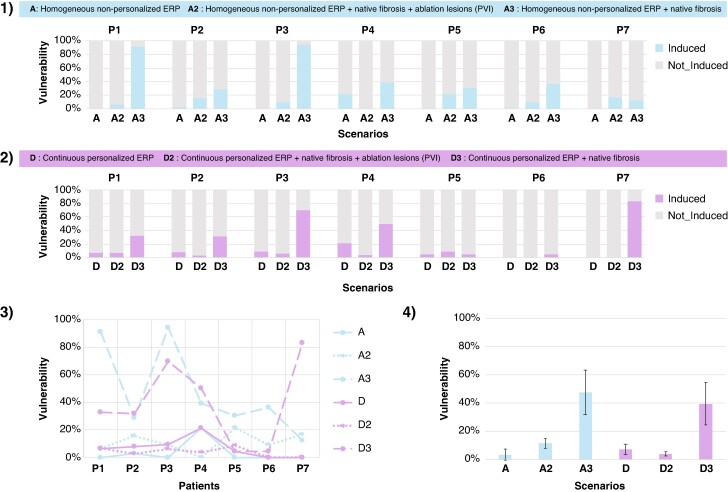
Comparison of fibrotic substrate modeling and effective refractory period personalization between scenario A (homogeneous non-personalized ERP) and scenario D (continuous with personalized ERP). *1*) Vulnerability for scenario A and two LVA substrate modeling scenarios: A2 with ablation lesions (*<*0.1 mV) and native fibrosis (*<*0.5 mV), and A3 as a state before PVI with only native fibrosis (*>*0.1 mV). *2*) Vulnerability for scenario D and two LVA substrate modeling scenarios: D2 with personalized continuous ERP with ablation lesions (*<*0.1 mV) and native fibrosis (*<*0.5 mV) and D3 as a state before PVI with personalized continuous ERP and only native fibrosis (*>*0.1 mV). *3*) Comparison of six scenarios for each patient. *4*) Mean vulnerability per scenario. Lines denote standard deviation. P1–P7 indicate individual patients. ERP, effective refractory period; LVA, low-voltage area.

We assessed the impact of incorporating fibrotic substrate informed by LVA into the models along with ERP personalization on arrhythmia vulnerability. Given that most patients had undergone previous PVI, we compared vulnerability with and without ablation lesions defined by regions where bipolar amplitude was *<* 0.1 mV. To avoid additional confounding factors, we compared scenario A (homogeneous non-personalized) and scenario D (continuous with personalized ERP) without fibrosis with their respective counterparts with fibrosis and ablation lesions A2 and D2 (*Figure 8*). Incorporating fibrosis and ablation lesions resulted in a vulnerability of 11.3 ± 7.3% and 3.9 ± 3.3% for A2 and D2, respectively. Incorporating only fibrosis without ablation lesions resulted in a vulnerability of 47.5 ± 32.0% and 39.4 ± 30.3% for A3 and D3, respectively. Area reduction due to PVI decreased vulnerability by 36.2% when comparing A3 vs. A2 (47.5 ± 32.0% vs. 11.3 ± 7.3%), and by 35.5% when comparing D3 vs. D2 (39.4 ± 30.3% vs. 3.9 ± 3.3%; *Figure 8*). The magnitude of the difference between A3 and D3 varied among patients, with some experiencing small differences in vulnerability, e.g. P2 and P4, while others showing a bigger difference, e.g. P1 and P7. On average, the homogeneously reduced ERP in A3 in the presence of native fibrosis without ablation lesions resulted in higher vulnerability compared with D3.

**Figure 9:**
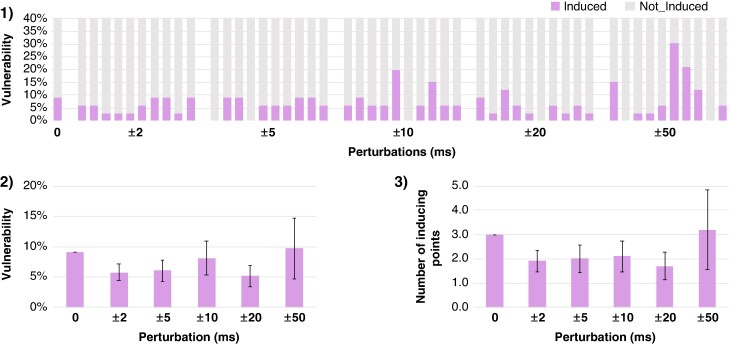
Sensitivity analysis for arrhythmia vulnerability of patient P3 comparing scenario D (personalized with continuous ERP) and perturbed ERP measurements. *1*) Vulnerability for each perturbation range. *2*) Mean vulnerability. *3*) Mean number of inducing points for each perturbation range. Lines indicate standard deviation. Perturbations of ±2, ± 5, ± 10, ± 20, and ±50 ms were randomly drawn, 10 times for each perturbation range, from a uniform distribution. The perturbations were incorporated to each measured ERP value and new interpolated continuous maps were generated from the perturbed ERP measurements. ERP, effective refractory period.

Incorporating perturbations to the measured ERP in the sensitivity analysis slightly impacted the vulnerability of the model from 9.1% in the baseline scenario D to 5.8 ± 2.7%, 6.1 ± 3.5%, 6.9 ± 3.7%, and 5.2 ± 3.5%, observed for perturbations in the range of ±2, ± 5, ± 10, and ±20 ms, respectively (*Figure 9*). Only when the perturbations were in the range of ±50 ms, a higher standard deviation was observed (9.7 ± 10.0%).

## Discussion

In this study, we assessed arrhythmia vulnerability in a cohort of seven patient-specific atrial models, each with information on the distribution of ERP and low-voltage substrate. We compared vulnerability across four ERP scenarios: non-personalized homogeneous (A), non-personalized heterogeneous (B), personalized regional (C), and personalized continuous (D) distribution of ERP without substrate. Secondly, we investigated the impact on vulnerability of the interaction between native fibrosis and ablation lesions with ERP. Thirdly, we conducted a sensitivity analysis to evaluate the effects of uncertainty in ERP measurements on arrhythmia vulnerability. The four main highlights of our study are as follows: (i) differences in arrhythmia vulnerability between personalized and non-personalized scenarios should be acknowledged, particularly for patients with low ERP; (ii) an increased dispersion of the ERP in personalized scenarios had a greater effect on re-entry dynamics than on mean vulnerability values; (iii) the incorporation of personalized ERP had a greater impact on inducibility than had a homogeneously reduced ERP; however, this effect reversed when native fibrosis was included, with a higher inducibility for the homogeneously reduced ERP scenario; and (iv) ERP measurement uncertainty up to 20 ms slightly influences arrhythmia vulnerability.

### Effect of effective refractory period personalization on arrhythmia vulnerability and dynamics

Personalized and non-personalized scenarios were different in the mean and dispersion of the ERP. Among all scenarios, the homogeneous non-personalized scenario A had the lowest vulnerability, while the regional personalized scenario C had the highest vulnerability. Heterogeneities in the form of regions in scenario C promote unidirectional blocks, thereby increasing vulnerability, while the homogeneous scenario A makes it less likely to induce re-entry even with a shorter ERP.^32^ Differences in vulnerability between personalized and non-personalized scenarios were greater in patients with lower ERP (*<*240 ms), corresponding to P1–P4, with a total of 56 inducing points. In contrast, the remaining three patients (P5–P7) were almost non-inducible, with only five inducing points in total. During cursory follow-up, two out of seven patients (P1 and P3) recurred with AF after 4 and 8 months, respectively. Decreased inducibility in P5–P7 could be attributed to the reduced effective atrial size.^33^ Thus, differences in vulnerability between personalized and non-personalized scenarios cannot be neglected, particularly for patients with low ERP.

The effect of ERP personalization becomes more evident when analyzing re-entry dynamics. There were no significant differences in the TCL of the non-personalized scenarios A and B (167.1 ± 12.6 ms vs. 158.4 ± 27.5 ms, *P* = 0.43), nor were there significant differences between the personalized scenarios C and D (265.2 ± 39.9 ms vs. 285.9 ± 77.3 ms, *P* = 0.31). However, personalized scenarios had significantly slower TCL compared with non-personalized scenarios (*P <* 0.001). This finding suggests that the increased dispersion of the ERP in the personalized scenarios has a greater effect on re-entry dynamics than on the absolute value of vulnerability.

### Increased effective refractory period dispersion is associated with higher arrhythmia vulnerability

Previous clinical and simulation studies have analyzed the effect of ERP and APD dispersion on arrhythmia vulnerability in patients with persistent and paroxysmal AF.^34−36,4^ Dispersion can be defined both spatially and temporally. Spatial dispersion refers to the difference between the maximum and minimum values of ERP measurements,^36^ while temporal dispersion refers to variation exceeding 5% from the baseline value.^35^ In the study of Narayan *et al.*,^35^ pacing-induced AF from either the PVs or high RA was always preceded by an increased temporal APD dispersion. In a cohort of 47 patients with paroxysmal AF, ERP was measured in five sites in both atria and a higher ERP dispersion was found to be the only clinical predictor of AF inducibility.^36^ An interesting finding of this study was that in patients with induced AF, ERP dispersion was similar in those with self-sustained and self-terminated AF. In another cohort of 22 patient-specific bi-atrial models without personalized ERP, where the substrate was modeled based on late gadolinium enhancement MRI by applying changes in anisotropy, conduction, and remodelled electrophysiology, the 13 models in which AF was induced had significantly larger APD gradients.^37^ In our results, scenarios with higher ERP dispersion in patients with a lower ERP mean had higher inducibility.

Previous studies have demonstrated that introducing a ± 10% homogeneous variation to the baseline APD increases uncertainty in both the quantity and preferred locations of re-entrant drivers.^38,39^ Consequently, it is expected that higher variations would also impact re-entry inducibility. In our results, clinical ERP measurements ranged from 222 to 295 ms, with a mean dispersion of 46.5 ± 15.8 ms corresponding to a mean variation of 18.0%. When compared with literature-based ERP values (157 ms for the LA), the variation between this value and the maximum observed clinical ERP reaches 87.9%. We conclude that increased inducibility depends on both reduced mean ERP and increased dispersion.

In this study, electrophysiological and substrate information was obtained through single-chamber electroanatomical mapping. A previous study from our group^40^ showed that arrhythmia vulnerability is higher in bi-atrial models than LA-only models. This increased vulnerability is due to exacerbated electrophysiological and substrate heterogeneity in the RA and the presence of interatrial connections. Effective refractory period measurements can typically only be obtained during the mapping procedure, which shortens the time frame available to build the personalized model and run the simulation to a few minutes. This warrants faster simulation approaches,^41^ reduced-order models or surrogate measurements of ERP distribution if no data from previous procedures can be utilized.

### Interaction between effective refractory period and substrate heterogeneities

In current clinical practice, it remains challenging to identify patients for whom PVI will be sufficient to prevent AF recurrence without additional ablation lesions. Six out of seven patients in our cohort had undergone prior PVI, indicating that PVI was ineffective in preventing AF recurrence. It is likely that substrate progression and gaps in PVI promoted AF recurrence.^42,43^ Regional heterogeneities in ERP dispersion are believed to be capable of sustaining AF on vulnerable substrates. Several atrial *in silico* studies have shown that fibrosis regions can anchor or block re-entrant drivers.^37,44–46^ Our results showed that the presence of fibrosis and ablation lesions had a higher impact on vulnerability than ERP. However, the combination of fibrotic substrate and ERP had different effects on vulnerability. Scenarios in which both native fibrosis and ablation lesions were considered (scenarios A2 and D2) had lower vulnerability compared with those having native fibrosis only. A possible explanation is the reduced effective atrial size due to PVI lesions.^33^ We tried to simulate a state prior to PVI in scenarios A3 and D3, although it is likely that native fibrosis based on the identification of regions with voltage *>*0.1 and *<*0.5 mV might not accurately represent the pre-ablation state. On average, the lower mean ERP in A3 in the presence of native fibrosis without ablation lesions resulted in higher vulnerability compared with a dispersed ERP distribution as in D3. As substrate areas have a significant impact on model inducibility, further studies should focus on providing a more detailed description of their spatial distribution for informing patient-specific models.

### Incorporating uncertainty to effective refractory period measurements

Measured ERP values depend on the time resolution of the S2 coupling interval, with higher resolution leading to more accurate values. To determine whether the addition of ERP perturbations would affect vulnerability, we conducted a sensitivity analysis by running 50 additional vulnerability assessments. Our results suggest that variations in the range of 2–20 ms did not markedly change the number of inducing points, and vulnerability remained similar, indicating that further reductions (<10 ms) in the S2 coupling interval would not impact model inducibility. Only when the perturbations were in the range of 50 ms, a higher standard deviation was observed. However, these differences in vulnerability might become more pronounced when functional substrate is incorporated. As mentioned before, re-entry dynamics are affected when ERP is personalized, rather than inducibility; therefore, future studies should assess the impact of ERP uncertainty together with substrate information on re-entry dynamics.

### Limitations

The small sample size can limit the generalization of our findings. No bi-atrial electrophysiological mapping data were available; therefore, single-chamber patient-specific models were generated, which did not allow for the assessment of ERP dispersion effects between the LA and RA on arrhythmia vulnerability. In the optimization process to adapt the cellular electrophysiology model to measured ERP, we reduced the dimensionality of the parameter set space by constraining the range of variation for each parameter in a linear fashion from normal healthy to changes due to persistent AF. We did not personalize CV distribution. The rate-dependent nature of ERP was not evaluated in our study as clinically measurements of the ERP were only obtained at 500 ms S1 cycle length.

## Conclusions

Incorporation of patient-specific ERP values affects the assessment of AF vulnerability. Differences in arrhythmia vulnerability between personalized and non-personalized scenarios should be acknowledged, particularly for patients with low ERP. An increased dispersion of the ERP in personalized scenarios had a greater effect on re-entry dynamics than on mean vulnerability values. The incorporation of personalized ERP had a greater impact on inducibility than had a homogeneously reduced ERP, with this effect reversing once fibrosis was included. ERP measurement uncertainty up to 20 ms slightly influences arrhythmia vulnerability. Functional personalization of atrial *in silico* models appears essential and warrants confirmation in larger cohorts.

## Translational perspective

Patient-specific atrial computer models are at a pivotal stage to demonstrate clinical applicability. Most patient-specific atrial models currently rely solely on structural information. This study evaluates the impact of incorporating functional information derived from clinically measured effective refractory period (ERP). The findings indicate that personalized ERP dispersion significantly influences re-entry dynamics and that the effects of personalized ERP on inducibility also depend on the presence of fibrosis. Incorporating functional personalization into models could improve the ability to stratify patients based on their individual characteristics, leading to more tailored and effective treatment strategies for atrial fibrillation.

## Supplementary Material

euae215_Supplementary_Data

## Data Availability

The data underlying this article including bilayer models, adjustment files, and source code to reproduce the simulated re-entries are publicly available at https://doi.org/10.5281/zenodo.10726677.^31^
